# Immunotherapy in patients with brain metastasis: advances and challenges for the treatment and the application of circulating biomarkers

**DOI:** 10.3389/fimmu.2023.1221113

**Published:** 2023-11-03

**Authors:** E. M. Brozos-Vázquez, C. Rodríguez-López, A. Cortegoso-Mosquera, S. López-Landrove, L. Muinelo-Romay, J. García-González, R. López-López, L. León-Mateos

**Affiliations:** ^1^ Medical Oncology Department, Complexo Hospitalario Universitario de Santiago de Compostela, Santiago de Compostela, Spain; ^2^ Medical Oncology Department, Complexo Hospitalario Universitario de A Coruña, Santiago de Compostela, Spain; ^3^ ONCOMET, Instituto de Investigación Sanitaria de Santiago, Santiago de Compostela, Spain; ^4^ CIBERONC, Madrid, Spain

**Keywords:** solid tumors, brain metastasis, biomarkers, immunotherapy, liquid biopsy

## Abstract

The central nervous system (CNS) is one of the most frequent metastatic sites of various cancers, including lung cancer, breast cancer and melanoma. The development of brain metastases requires a specific therapeutic approach and is associated with high mortality and morbidity in cancer patients. Advances in precision medicine and the introduction in recent years of new drugs, such as immunotherapy, have made it possible to improve the prognosis of these patients by improving survival and quality of life. New diagnostic techniques such as liquid biopsy allow real-time monitoring of tumor evolution, providing molecular information on prognostic and predictive biomarkers of response to treatment in blood or other fluids. In this review, we perform an exhaustive update of the clinical trials that demonstrate the utility of immunotherapy in patients with brain metastases and the potential of circulating biomarkers to improving the results of efficacy and toxicity in this subgroup of patients.

## 1 Clinical challenge

The brain is one of the most frequent sites of metastases from some types of solid tumors, such as lung, breast cancers and melanoma. The presence of disease in the brain represents a dismal prognosis and devastating complications for the quality of life of oncological patients and can lead to a different evolution in relation to the histological and molecular type, and clinical characteristics of the tumor. Recent advances in Oncology have improved survival rates, with the incidence of brain metastases now estimated at around 20%-40% (1, 2). Actually, in recent years overall survival in these cancers has increased significantly due to the introduction of new drugs, including immunotherapy. This treatment has been used in routine clinical practice since phase III clinical trials have demonstrated its efficacy in advanced tumors such as lung cancer (3, 4) or melanoma (5, 6). However, there is no extensive literature on the subgroup of patients with cerebral or leptomeningeal disease because these patients have traditionally been excluded from the clinical trials.

The mainstays of the treatment in cases with secondary brain involvement are surgery and stereotactic radiosurgery if the patient has a single brain metastasis (BM) or limited number; and radiotherapy and symptomatic management with corticosteroids and/or antiepileptic drugs on patients with extensive number or volume of brain lesions. Classical chemotherapy has played a limited role due to its limited passage through the blood-brain barrier. However, targeted therapy is effective in the presence of onco-addictive molecular alterations, and immunotherapy is nowadays crucial in the landscape treatment of lung cancer and melanoma.

Moreover, we already know the fact that a drug could be effective in controlling metastatic disease at the systemic level, but not necessarily imply that it is active on synchronous disease in the central nervous system (CNS). On the other hand, the genetic characteristics of the primary tumor and different metastatic sites might differ (7, 8), and the response to the same treatment may therefore be different. Genetic, epigenetic, and transcriptomic changes in brain metastases lead to changes in the tumor microenvironment, making this disease immunologically more inactive with respect to the primary tumor (9–11).

Besides, there is an unmet clinical need for discovering biomarkers that can help to predict a high risk of brain metastases in certain tumor types, as well as to individualize management once they are present. We have the GPA (Graded Prognostic Assessment) prognostic tool whose objective is to estimate survival in patients with lung cancer based on clinical variables, without considering molecular factors, which nowadays must be considered due to the demonstrated relevance of molecular biology in cancer (12). Given the complexity of obtaining a biopsy of brain tissue, the development of non-invasive biomarkers is of high relevance in the context of secondary brain lesions.

The aim of this review is to analyze published data on the efficacy of immunotherapy as a treatment for patients with metastatic brain disease, focusing on lung, breast, and melanoma tumors. We make an overview of the most relevant clinical trials performed to explore the efficacy of immunotherapy to treat brain metastasis and comment on the current knowledge and the potential of liquid biopsy-based biomarkers to predict the therapy response and monitor the disease evolution.

## 2 Immunotherapy in lung cancer patients with brain dissemination

Lung cancer (LC) is a heterogeneous disease, histologically and molecularly (13, 14). There are two main subtypes of LC, small cell lung carcinoma (NSCLC) and small cell lung carcinoma (SCLC), accounting for 76% and 13%, respectively. LC remains the most common cause of cancer-related death (15). However, in the last decade, mortality associated with NSCLC has decreased due to a reduction in the incidence of LC and an increase in the overall survival of patients with NSCLC (16). Furthermore, the improvement in LC survival is related to a deeper understanding of the genomic profile of NSCLC that has allowed progress in LC treatment in two directions: on the one hand, the introduction of targeted therapies against mutations in driver oncogenes, and, on the other hand, immune checkpoint inhibitors (ICIs) (16).

### 2.1 Non-small cell lung cancer

BMs are common in patients with advanced NSCLC and occur in 20-30% of patients (17, 18). Currently, treatment of cerebral disease is based on surgery, holocranial radiotherapy (WBRT) or stereotactic radiosurgery (SBRT) (19, 20). However, this practice is changing in patients with NSCLC and driver oncogene mutations, as tyrosine kinase inhibitors have shown good CNS penetrance and a high response rate of intracranial disease (21–24).

Immunotherapy with inhibitors against the programmed death 1 (PD-1) receptor, its ligand (PD-L1) or cytotoxic T-lymphocyte-associated protein 4 (CTLA-4) has revolutionized the therapeutic scenario for patients with advanced NSCLC (aNSCLC) without driver mutations (25). Today, the current therapeutic approach to an individual patient with aNSCLC is based on tumor expression of PD-L1. Thus, in tumors with 50% PD-L1 expression, the use of ICIs as monotherapy is an effective option (26–28). While the combination of ICI plus platinum-based chemotherapy has been shown to improve efficacy parameters regardless of the percentage of PD-L1 expression (29–32). Although most of the clinical trials in this setting allowed the inclusion of patients with pre-treated and clinically asymptomatic CNS metastases, there are few data about the efficacy of the immunotherapy at the CNS level (**Table 1**).

**Table 1:** Outcomes of ICI in the treatment of NSCLC with basal BMs.

Author	Study type	Treatment	No patients with BMs	Outcomes
**Ready, et al.** (33)	Phase IIIB	Nivolumab plus Ipilimumab	49	mOS: 12.8 m 3-years OS rate: 21.0% mPFS: 2. 8 m 3-years PFS rate: 14.2%
**Reck, et al.** (34)	Phase III	Nivolumab plus Ipilimumab vs chemotherapy	68 66	mOS: 17.4 vs 13.7 m 5-year OS rates: 20% vs 6% 5-year intracranial PFS rates: 16% vs 6%
**Carbone, et al.** (35)	Phase III	Nivolumab plus ipilimumab and chemotherapy vs chemotherapy	51 50	mOS: 19.3 vs 6.8 m 2-year OS rates: 35% vs 12% mPFS: 10.6 VS 4.1 m
**Nadal, et al.** (36)	Phase II	Atezolizumab plus chemotherapy, followed by maintenance with pemetrexed plus atezolizumab	40	mOS: 13.6 m 2-year OS rate: 30.5% mPFS: 8.9 m Intracraneal mPFS: 6.9 m
**Hou, et al.** (37)	Phase II	Camrelizumab plus chemotherapy, followed by maintenance with camrelizumab and pemetrexed	45	mOS: 21.0 m mPFS: 7.4 m Intracraneal mPFS: 7.6 m
**Powell, et al.** (38)	Pooled analysis	Pembrolizumab plus chemotherapy vs chemotherapy	171	mOS: 18,8 m vs 7,6 m mPFS: 6,9 m vs 4,1 m
**Ozguroglu, et al.** (39)	Phase III	Cemiplimab vs chemotherapy	68	mOS: 18.7 vs 11.7 mPFS: 10.4 vs 5.3
**Goldberg, et al.** (40)	Phase II	Pembrolizumab	42	In patients with NSCLC and PD-L1 ≥ 1%: • mPFS 1.9 m • mOS 9.9 m • 2-years OS ratio 34%
**Mansfield, et al.** (41)	Pooled analysis	Pembrolizumab vs chemotherapy	293	Pts with NSCLC and PD-L1 ≥ 1%: • mOS: 13,4 vs 10,3 m Pts with NSCLC and PD-L1 ≥50%: • mOS: 19,7 vs 9,7 m

BMs, brain metastasis; m, months; mOS, median overall survival; mPFS, median progression-free survival; NSCLC, non-small cell lung cancer; Pts, patients.

In the CheckMate 817 clinical trial (33), the efficacy of nivolumab plus ipilimumab was prospectively evaluated in first-line treatment of special populations with NSCLC, including 49 patients with untreated brain metastases. In this subgroup the median OS was 12.8 months, with a 3-year survival rate of 21%. In addition, the median PFS was 2.8 months, with a 3-year PFS rate of 14.2% (42). To reinforce these data, *post-hoc* analysis data with a minimum five-year follow-up of patients with baseline brain disease included in the CheckMate 227 (Part 1) clinical trial have recently been published (34). In this population, the treatment with the combination of nivolumab plus ipilimumab prolongs OS versus treatment with platinum-based chemotherapy in those patients with CNS metastases at baseline. In addition, less patients with cerebral disease at the inclusion developed new brain metastases with nivolumab and ipilimumab versus chemotherapy (4% and 20%, respectively) (34).

Following this treatment rationale, the nivolumab plus ipilimumab combo was combined with 2 cycles of platinum-based chemotherapy in the CheckMate 9LA clinical trial, which included 101 patients with brain metastases (35). In a *post-hoc* study of this population, the combination of nivolumab plus ipilimumab chemotherapy showed a hazard ratio for the risk of death of 0.43, with a median OS of 19. 3 months versus 6.8 months in patients treated with chemotherapy alone; plus, a 2-year alive rate of 35% (35).

The multicenter phase II Atezo-BRAIN trial evaluated the efficacy and safety of combining atezolizumab with 4 or 6 cycles of carboplatin plus pemetrexed followed by maintenance with atezolizumab plus pemetrexed in patients with non-squamous NSCLC and CNS metastases (36). In this study, 40% of patients had confirmed intracranial response based on RANO-BM criteria (12 partial responses; 4 complete responses) and 19 (47.5%) patients achieved systemic response (all partial responses). No differences in the overall response rate in systemic and intracranial were observed according to PD-L1 expression or corticosteroid use at baseline. With a longer follow-up of 20 months, the median systemic PFS was 8.9 months and the median intracranial PFS was 6.9 months. In addition, the median OS was 13.6 and the estimated 2-year OS rate was 30.5%. OS was explored as a function of PD-L1 expression; thus, the median OS was higher for PD-L1 positive patients at 16.2 months compared to PD-L1 negative patients 10.7 months. However, these differences were not statistically significant due to limited statistical power. There was also no significant difference in OS between those who did and did not receive baseline dexamethasone treatment.

The clinical activity and safety of camrelizumab plus chemotherapy as first-line treatment in patients with advanced NSCLC with brain metastases was evaluated in a study carried out in Chinese population (37). The objective response rate in the intracranial disease was 52.5% in those patients that have at least one post-baseline tumor assessment and 46.7% in the full analysis. On the other hand, the objective response rate in the extracranial tumor burden was 47.5% and 42.2%, respectively.

Strengthening the efficacy of the combined treatment of ICI plus platinum-based chemotherapy are data from a pooled analysis of the clinical trials KEYNOTE-021, -189 and -407 (38). In patients with brain metastases, the median overall survival was 18.8 months with pembrolizumab plus chemotherapy and 7.6 months with chemotherapy with a 52% reduction in the risk of death. Median PFS was longer with pembrolizumab plus chemotherapy versus chemotherapy in patients with cerebral lesions (6.9 months versus 4.1 months). Finally, pembrolizumab with chemotherapy reached a higher objective response rate, regardless of BM status, if we compare it with chemotherapy alone.

The EMPOWER-Lung 1 trial assessed the efficacy and safety of cemiplimab in frontline advanced NSCLC with PD-L1 expression ≥50% and included 68 (12.1%) treated brain metastases patients (39). In this subgroup, analysis of PFS and OS showed that patients significantly benefited from cemiplimab compared to platinum-based chemotherapy.

On the other hand, Goldberg et al. (40), analyzed the efficacy of pembrolizumab in two cohorts of patients with NSCLC and CNS metastases: cohort 1 included 37 patients with PD-L1 expression ≥ 1%, while cohort 2 included 5 patients with PD-L1 expression <1%. No responses were observed in patients in cohort 2. In contrast, in cohort 1 the brain response rate was 29.7%. In addition, the overall response rate was 18.9%, considering both systemic and brain disease. A discordant response was observed in 6 patients. In this work the median PFS was 1.9 months and 33% of patients had not progressed to brain level at 12 months. Finally, the median OS was 9.9 months, and the 2-year alive rate was 34%. Importantly, in a pooled analysis of four studies, pembrolizumab was shown to reduce the risk of death by 17% compared to chemotherapy in patients with baseline brain metastases and NSCLC with PD-L1 expression >1% (41). Thus, in this population the median OS was 13.4 and 10.3 months, respectively. Furthermore, this benefit appears greater in patients with NSCLC and PD-L1 TPS expression ≥50% with brain metastases at baseline, with a 33% reduction in risk of death; median OS was 19.7 and 9.7 months, respectively. In addition, pembrolizumab provided similar results in patients with brain metastases or without them.

Globally, although limited data are available on the role of immunotherapy in NSCLC and brain metastases, ICIs alone or in combination with chemotherapy have shown promising intracranial clinical activity and safety outcomes (43). Nevertheless, the mechanism of how ICIs act in the brain niche and their interaction with the tumor microenvironment is unknown. Therefore, characterizing the immune phenotype of cerebral disease and better understanding the relation with the immune cells, resident stromal cells along with neoplastic cells are crucial to improve the results of immunotherapy in our patients.

### 2.2 Small cell lung cancer

SCLC, a highly aggressive type of lung cancer, represents 15% of all lung cancer cases and is strongly linked to tobacco use. Regrettably, patients diagnosed with extensive disease SCLC (ED-SCLC) face a mere 2% 5-year survival rate (44–49). While chemotherapy has long been the established treatment approach for SCLC, recent advancements have introduced alternatives like immunotherapy, which have shown potential in enhancing survival rates (45–48). Nonetheless, a significant portion of ED-SCLC patients do not experience the benefits of this innovative treatment (49).

Brain metastases pose a significant threat to the well-being and survival of individuals with SCLC, leading to notable morbidity and mortality while greatly impacting their quality of life (50). The diagnosis of brain metastases originating from SCLC relies on imaging techniques like CT scans and MRI scans. Research indicates that around 10-14% of SCLC patients already have brain metastases at the time of diagnosis, and during the course of the disease, this number can rise to 50% (50). The incidence of brain metastases is higher in patients with extensive-stage SCLC (ES-SCLC) compared to those with limited stage, LS-SCLC. Typically, the median survival for individuals with brain metastases from SCLC is approximately 6 months, although there have been instances of patients surviving beyond one year. Factors that contribute to improved survival rates include younger age, absence of tumor growth, and a favorable performance status (50).

The standard treatment approach for brain metastases originating from SCLC commonly entails a combination of radiation therapy, chemotherapy, and immunotherapy (50, 51). Due to the aggressive nature of the disease, surgical intervention is seldom employed (51). Historically, radiotherapy has been utilized as a preventive measure to impede the development of metastases in cases of thoracic-confined or metastatic disease (known as prophylactic cranial irradiation) (51). Additionally, radiotherapy is administered as a therapeutic intervention when there is visible macroscopic disease. Chemotherapy, often comprising platinum agents and etoposide, is employed with or without immunotherapy to diminish the risk of recurrence and manage symptoms (51).

Of note, SCLC can be categorized into four subtypes named SCLC-A, SCLC-N, SCLC-P, and SCLC-Y, based on the expression of specific genes, namely achaete-scute homolog 1 (*ASCL1*), neurogenic differentiation factor 1 (*NeuroD1*), POU class 2 homeobox 3 (*POU2F3*), and yes-associated protein-1 (*YAP-1*), respectively (52, 53). Research studies have highlighted the significance of recruiting and activating T cells at the tumor sites as crucial steps in cancer immunotherapy. A subsequent study indicated that *YAP-1* could serve as a prognostic marker for T cell-induced inflammatory responses (52). The study findings demonstrated an inverse correlation between *YAP-1* and the activation and differentiation of CD4^+^ and CD8^+^ T cells, presenting significant potential for the immunotherapy of LS-SCLC (52). Additionally, the SCLC-Y subtype exhibits a notable inclination towards undergoing epithelial-to-mesenchymal transition (EMT), immune cell infiltration, and heightened antigen presentation capacity (53). This particular SCLC subtype demonstrates relatively higher sensitivity to immunotherapy and combination chemo-immune therapy.

Xiao et al. conducted a meta-analysis aiming to assess the outcomes of lung cancer patients who received immunotherapy alone or in combination with other treatments such as chemotherapy, targeted therapy, or radiotherapy (54). The analysis focused on phase 2 or 3 randomized controlled trials. Additionally, they compared chemotherapy or placebo and examined hazard ratios for overall survival. The meta-analysis revealed a pooled interaction of 0.72 for non-small cell lung cancer (NSCLC) and 1.41 for small cell lung cancer (SCLC) in patients with and without brain metastases. This difference was indicated by a heterogeneity test of interaction between the two subgroups (54).

In the CASPIAN trial, the combination of durvalumab with the etoposide-platinum (EP) regimen was investigated for the treatment of patients with extensive-stage SCLC (55). The trial demonstrated a median overall survival (OS) of 13 months, which was 2.7 months longer than the control group. The study included patients with brain metastases, either receiving treatment or without symptoms in the central nervous system (CNS) (10% in the control group and 10.4% in the monoclonal antibody group). The results indicated that the combination of monoclonal antibody and chemotherapy regimen delayed intracranial progression, reduced the need for brain radiotherapy, and provided certain benefits in terms of improving progression-free survival (PFS) and OS in patients with brain metastases.

The IMpower 133 trial, a large global phase III clinical trial, aimed to evaluate the effectiveness of atezolizumab in combination with chemotherapy as a first-line treatment for metastatic SCLC. The trial results demonstrated a statistically significant 2-month improvement in overall survival (p=0.015) for this subgroup of patients (47). However, only 8.7% of patients with CNS metastasis were included in this trial, making it inconclusive regarding the benefits of atezolizumab for patients with BM (56). On the other hand, the KEYNOTE-604 trial, a double-blind, placebo-controlled phase III trial, enrolled previously untreated patients with extensive-stage SCLC (ES-SCLC) (49). These participants were randomly assigned to receive EP (etoposide-platinum) chemotherapy with or without pembrolizumab. The median overall survival (OS) for the experimental group was 10.8 months, while the control group had a median OS of 9.7 months. The difference between the two groups was statistically significant (P = 0.0164). However, the confidence interval (CI) values for subgroups with fewer than three metastatic sites and brain metastases were wide and overlapped with the total population. Consequently, more patients with BM need to be included in studies to accurately assess the efficacy of pembrolizumab in this particular scenario.

The genomic profile of SCLC is characterized by extensive chromosomal rearrangements and a high mutational burden, including the inactivation of tumor suppressor genes *TP53* and *RB1* (57). However, there are currently no validated predictive biomarkers available to guide treatment decisions or stratify patients (58). Liquid biopsies, such as circulating tumor DNA (ctDNA) and circulating tumor cells (CTCs), show promise as alternative methods to guide the therapy selection and the follow-up (58–60).

## 3 Immunotherapy in breast cancer patients with brain dissemination

Breast cancer is one of the most prevalent tumors in the world and the one that most frequently metastasizes to the central nervous system (CNS), being the second most common cause of brain metastases after lung carcinoma and above melanoma. Between 15-30% of patients with advanced breast carcinoma will develop CNS disease, which will have prognostic implications, limiting survival to around 15 months, as well as a major impact on their quality of life due to the neurological complications that these patients develop (61, 62).

There are some histological subtypes with molecular features that determine a different biological behavior and natural history of disease. HER2^+^ and triple negative (TNBC) are the ones that most frequently cause BM. For the appropriate management of this disease, we have the added difficulty that patients with CNS disease are often excluded from clinical trials, so there is less evidence available. Currently, the recommended treatment of brain metastatic breast cancer disease (BCBM) includes locoregional therapy and systemic therapy, or a combination of both. In the case of HER2^+^ BC, different drugs directed against the HER2 receptor have proven to be effective in the treatment of advanced disease (63), including cerebral disease, sometimes avoiding aggressive surgery or brain irradiation with medium and long-term consequences.

In recent years, systemic therapies for the treatment of advanced TNBC have included ICIs such as atezolizumab and pembrolizumab, in combination with chemotherapy, for patients whose tumor expresses PD-L1, quantified by IHC with non-homogeneous methods in different studies. In these studies, exclusion criteria have prevented recruitment of patients with BM. Therefore, we do not have useful biomarkers of response to immunotherapy in liquid biopsy in metastatic brain disease due to breast carcinoma which are valid for use in routine clinical practice. From retrospective literature studies that obtain information from different genetic databases to perform differential expression analysis between primary breast tumors and BM, some genes emerge as possible subjects of future research to test their use as potential biomarkers predictive of drug response or therapeutic targets (64, 65).

A study published by Lu et al. found increased plasma cell infiltration in BM and decreased infiltration by M2 macrophages, as well as overexpression of the immuno-related genes *THY1* and *NEU2*, proposing them as possible therapeutic targets (7). The *ARG2* expression, which has an immunosuppressive role, appears to be higher in BCBM, indicative of a lower T-cell mediated response (10). In terms of epigenetic alterations, HLA-A methylation has typically been identified in metastatic TNBC disease, resulting in decreased expression of HLA-A and associated immune cells, which may be a biomarker of unresponsiveness to ICIs as well as a therapeutic target, using DNA demethylating drugs in combination (11). The signature Macrophage receptor with collagenous structure (MARCO) has been linked to prognosis, immune cell infiltration and ICI treatment in different tumors, and is a potential biomarker that still needs to be further explored (66).

There are some approaches about molecular development in this subpopulation of patients (67). An ongoing phase II investigates the efficacy of different combinations depending on molecular subtype; the cohort of TN patients receive bevacizumab, SHR1316 (a novel anti-PD-L1) and platinum (NCT04303988). Two studies explore the efficacy of immunotherapy in combination with radiosurgery (SRS), a phase I/II study with pembrolizumab in BCBM (NCT03449238), and a phase II study with atezolizumab in TNBM (NCT03483012). The use of other ICIs, such as ipilimumab (anti-CTLA4), are being tested in advanced TNBC, so far without high efficacy data and without specific development in cases of brain disease. Other newer approaches emerging in the field of immunotherapy that are being explored in the HER2^+^ subtype, such as the use of CAR-T, are also lacking research in this setting of BM disease by triple-negative subtype.

Some ongoing clinical trials are evaluating the efficacy of immunotherapy in combination with other drugs or radiotherapy in patients with BCBM depending on molecular subtype. The use of other ICIs, such as ipilimumab (anti-CTLA4), are being tested in advanced TNBC, so far without high efficacy data and without specific development in cases of brain disease. Other newer approaches emerging in the field of immunotherapy that are being explored in the HER2^+^ subtype, such as the use of CAR-T, are also lacking research in this setting of metastatic brain disease by triple-negative subtype.

## 4 Immunotherapy in melanoma patients with brain dissemination

Melanoma is the third most common type of cancer, after breast and lung cancer, to metastasize to CNS. Between 40% and 50% of patients diagnosed with melanoma will generate clinically detectable CNS metastases during the course of the disease (68). Prior to the development of new targeted therapies and immunotherapy, the presence of BM in melanoma was associated with poor prognosis, with a median overall survival of 4 months (68, 69). Leptomeningeal involvement, the size and number of BM, the presence or absence of neurological symptoms and mutational status are different prognostic factors that affect the survival of these patients (69).

Historically, treatments for melanoma with CNS metastases consisted of surgery and radiotherapy, with poor results and short survival. In the last 10 years, with the emergence of targeted therapies (BRAF inhibitors and MEK inhibitors) and the efficacy of immunotherapy in this context, the prognosis of these patients has changed. The *BRAF* V600 mutation is present in 40-50% of metastatic melanomas and leads to activation of MAPK and ERK pathways. In a study of matched samples of primary melanoma tumors and BM, a mutation concordance of 80% was observed (70). The presence of *NRAS* and *C-KIT* mutation in patients with melanoma and BM was 22% and 11% respectively (71). Loss of PTEN function has been detected in about 10-30% of melanomas, most frequently in *BRAF-*mutated tumors (71). Activating mutations in *AKT1* and *AKT3* are rare (1-2%) (71). In studies of paired biopsy samples from extracranial and CNS metastases, a similar mutation profile tends to be observed. As an exception, BM express specific molecular features in the PI3K/AKT pathway (72). Melanoma CNS metastases showed increased expression of various protein markers that activate the PI3K/AKT pathway compared to extracranial disease, being a potential therapeutic target (72).

In the melanoma population with BM and *BRAF* mutated tumors, the combination of immunotherapy with BRAF and MEK inhibitors has been tested in recent years, as well as the sequencing of these treatments. Data have recently been published from the TRICOTEL clinical trial, a phase II study with 2 cohorts patients diagnosed with melanoma and BM: one cohort without *BRAF* V600 mutated tumors, and another one with *BRAF* V600 mutated ones (73). The cohort *BRAF* negative was treated with atezolizumab and cobimetinib. The other group was treated with atezolizumab, cobimetinib and vemurafenib. Sixty-five patients were enrolled in the study. The intracranial objective response rate was 42% in the *BRAF* V600 mutation-positive cohort and 27% in the non-mutated population. Another study also analyzed the time to develop central nervous system metastases in patients treated with atezolizumab or placebo plus vemurafenib and cobimetinib (74). In the 514 patients included in the immunotherapy and targeted therapy cohort, 25% of patients developed brain metastases with a follow-up of 29.8 months, and 28% in the placebo group with a follow-up of 22.8 months. The time to onset of first CNS metastases was later in the atezolizumab, vemurafenib and cobimetinib group, which is interesting because represents some kind of activity in patients that are kind to generate brain metastases.

Several clinical trials assessed the efficacy of immunotherapy specifically in patients with melanoma and intracranial metastases. One of the first studies published in this context evaluates the role of Ipilimumab (Anti-CTLA-4) (75). This phase II study included patients in cohort A, neurologically asymptomatic and without corticosteroid therapy; and cohort B, symptomatic and on stable doses of corticosteroids. They received 4 doses of ipilimumab every 3 weeks, followed by 1 dose every 12 weeks if clinically stable. Disease control measuring the intracranial metastases was achieved in 24% of patients in cohort A and in 10% of cohort B patients. Extracranial disease control was 27% and 5% respectively. The different outcomes of the cohorts were related to patient characteristics, with cohort B being a poorer prognostic group and to the negative effects of corticosteroid therapy on immunotherapy. In the NIBIT-M1 phase II clinical trial, they combined treatment with ipilimumab and fotemustine, since chemotherapy-induced release of tumor antigens could amplify the anti-tumor activity of immunotherapy (76). Of the 20 patients included in the study, 50% had disease control, showing benefit if compared with historical cohort of patients with these features.

The efficacy of treatment with pembrolizumab and nivolumab monotherapy was also studied in the context of patients with BM. In a phase II study with patients affected by melanoma or untreated NSCLC adenocarcinoma with CNS metastases the activity of pembrolizumab was interrogated (77). Of the 18 melanoma patients included, response of BM was achieved in four patients. In another phase II clinical trial, 23 patients with untreated asymptomatic melanoma and BM were included without the need for corticosteroids. Seventy percent of the patients had received prior systemic therapy (78). Twenty-six percent of patients had a brain response, which was concordant with the systemic response. Median PFS was 2 months and OS was 17 months.

In clinical trials using immunotherapy in monotherapy, intracranial objective response rates were 16% with ipilimumab, 26% with Pembrolizumab and 20% with nivolumab (75–80). The combination of nivolumab and ipilimumab was investigated in two separate studies for patients with melanoma BM (79, 80). In the Australian study (ABC), asymptomatic patients without prior local treatment were assigned to nivolumab plus ipilimumab 4 doses every 3 weeks, followed by nivolumab every 2 weeks (Cohort A), or nivolumab every 2 weeks (Cohort B and C) (80). Cohort C included patients with failure of local therapy, with neurological symptoms or leptomeningeal disease, to receive nivolumab monotherapy. The best intracranial response at 12 weeks or later was 51%, 20% and 6% respectively in each cohort. PFS at 24 months was 49%, 15% and 6%, respectively. OS at 24 months was 63%, 51% and 19% respectively. The phase II CheckMate 204 study enrolled asymptomatic patients to receive nivolumab and ipilimumab every 3 weeks, 4 doses, followed by nivolumab every 2 weeks (79). The intracranial clinical benefit rate (defined as the percentage of patients had stable disease at 6 months, complete or partial response) was 57%, with 26% complete responses. The combination seemed to prevent intracranial progression for more than 6 months in 64% of patients (150). The treatment with ipilimumab and nivolumab achieved higher intracranial response rates in the different clinical trials compared to anti-PDL1 monotherapy (nivolumab or pembrolizumab) at the cost of higher toxicity (**Table 2**).

**Table 2:** Clinical Trials with immunotherapy in patients with melanoma and BM.

Author	Study type	Treatment	No patients with BMs	Outcomes
Margolin, et al. (75)	Phase II	Ipilimumab	Cohort A 51	IDCR: 25% mOS: 7.0 m mPFS: 2.7 m Intracraneal mPFS: 1.9 m
			Cohort B^#^ 21	IDCR: 10% mOS: 3.7 m mPFS: 1.3 m Intracraneal mPFS: 1.2 m
Di Giacomo, et al. (76)	Phase II	Ipilimumab and Fotemustine	20	IDCR: 50.0% mOS: 13.4 m mPFS: 4.5 m Intracraneal mPFS: 3 m
Goldberg, et al. (77)	Phase II	Pembrolizumab	18	IRR: 22%
Kluger, et al. (78)	Phase II	Pembrolizumab	23	IRR: 26% mOS: 17 m mPFS: 2 m Intracraneal mPFS: 3 m
Long, et al. (80)	Phase II	Nivolumab plus Ipilimumab, followed by Nivolumab	Cohort A 35	IRR 46% mOS: NR mPFS: 13.8 m Intracraneal mPFS: NR
		Nivolumab	Cohort B 25	IRR 20% mOS: 13.8 m mPFS: 2.6 m Intracraneal mPFS: 2.6 m
		Nivolumab	Cohort C^#^ 16	IRR 6% mOS: 5.1 m mPFS: 2.6 m Intracraneal mPFS: 2.3 m
Tawbi, et al. (79)	Phase II	Nivolumab plus Ipilimumab, followed by Nivolumab	94	IDCR: 57% 1-year OS rate: 81.5% 1-year PFS rate: 56.6% 1-year intracraneal PFS rate: 59.5%

IDCR, intracranial disease control rate; CR, complete response; PR, partial response; SD, stable disease. NR, Not reached; PFS, Progression Free Survival; OS, Overall Survival.

^#^Patients neurologically symptomatic.

## 5 Liquid biopsy to characterize brain metastases and guide immunotherapy

There is a clear interest in addressing the study of liquid biopsy in tumors that have metastasized to the brain. Surgery, in patients with BM located in eloquent or deep brain area, could be difficult and may cause neurological sequelae. Therefore, the possibility of characterizing the disease non-invasively represents a window of opportunity to guide the diagnosis, treatment, and monitoring of the therapeutic response when there is brain dissemination. Likewise, the focal study of a brain lesion by traditional biopsy may not fully capture intertumoral heterogeneity (81). In addition, it is known that BM may have different genomic alterations than primary extracranial tumors, and it is important to analyze genomic alterations specific to brain lesions in order to select the optimal therapeutic approach (72). To overcome these limitations, the study of circulating markers provides us with a more global vision of the molecular features of different regions within the same lesion and the possibility of approaching the study of BM in a non-invasive way (82).

**Figure 1:**
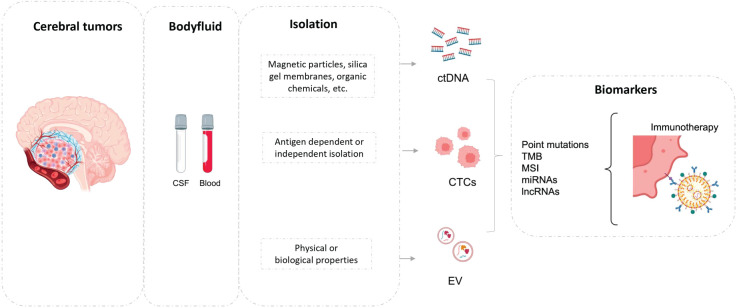
Brain metastases shed to blood or CSF, and biomarkers could be studied (ctDNA, CTCs or EV). CSF, cerebrospinal fluid; ctDNA, circulating tumoral DNA; EV, extracellular vesicles.

In the era of precision oncology, the ability to analyze liquid biopsies represents an important element to guide the selection of the treatment and supervise tumor evolution in real time. Any biofluid containing tumor material that can be molecularly characterized, represents a liquid biopsy. This term therefore includes blood, but also other corporal fluids like saliva, urine, bile, pleural effusion, or cerebrospinal fluid, among others. The tumor material present in these biofluids consists mainly of circulating tumor cells (CTCs), nucleic acids (cNAs) including DNA and RNA, and circulating extracellular vesicles (cEVs). Through the characterization of these circulating elements, we can obtain valuable information on mechanisms that favor tumors dissemination and progression, also in the context of brain lesions (82) (**Figure 1**). Knowledge about the application of each of these tumor elements in the context of BM is described in more detail below.

### 5.1 Circulating tumor DNA

Free tumor DNA can be released from primary tumor cells, CTCs, micrometastases, or macrometastases. Most of this DNA comes from tumor cells that enter apoptosis or necrosis and release their DNA with varying degrees of fragmentation into the bloodstream. The fraction of ctDNA in the total free circulating DNA (cfDNA) released by healthy cells is usually low, less than 1%, but can vary from less than 0.1% to more than 90% (83). ctDNA is characterized by the presence of somatic genetic alterations such as SNVs (single-nucleotide variants), CNVs (copy number variants) or Indels, as well as a tendency to be more fragmented, showing sizes that ranks from 90 to 170 base pairs (84). ctDNA is also known to reflect the epigenetic signatures of nuclear DNA (85, 86).

In order to detect ctDNA, it is necessary to apply highly sensitive techniques that can target a small panel of mutations such as digital PCR or BEAMing or cover a large panel of genes and even the whole exome/genome (NGS, Next-generation Sequencing; WGS, Whole-genome sequencing, or WES, Whole-exome sequencing). Generally, higher genome coverage is associated with lower sensitivity of the analysis (87).

There is already a great deal of evidence on the value of cfDNA analysis to genotype advanced stage tumors for personalized therapy selection, but also as a tool to monitor response to treatment or detect the presence of minimal residual disease (MRD) post-surgery and guide adjuvant treatment. In fact, the FDA (Food and Drug Administration) has approved several diagnostic tests to detect *EGFR* or *PIK3CA* mutations in lung and breast tumors. The FoundationOne Liquid CDx and Guardant360 liquid CDx panels (using free circulating DNA, cfDNA) have also recently been approved as companion diagnostic tests for several targeted therapies and immunotherapy (88). In the context of immunotherapy treatment, numerous studies have demonstrated the possibility of analyzing both tumor mutational rate and microsatellite instability (MSI) status in circulating free DNA samples, two biomarkers that predict response to checkpoint inhibitors (89). On the other hand, TMB has traditionally been tested in tumor tissue samples. However, Gandara et al. reported an analysis of TMB by NGS in a subgroup of patients from the POPLAR and OAK studies, comparing tumor sample with pre-treatment plasma in the same patient cohort (90). A positive correlation (0.64 Spearman rank) was found between tissue and blood sample, so that plasma TMB (bTMB) by liquid biopsy is considered a predictive biomarker of progression-free survival in patients treated with Atezolizumab monotherapy, when the value is higher than 16 mutations per megabase, as a clinically meaningful and technically robust threshold (90). A biomarker analysis with bTMB was also conducted in the phase III MYSTIC study, demonstrating its association with OS, PFS and ORR in patients treated with ICIs (91).

Although blood is the most used fluid for the study of cfDNA, several studies have shown that the concentration of tumor free DNA in brain and central nervous system lesions is higher in cerebrospinal fluid (CSF), which could be the preferred biofluid for the molecular characterization of these lesions, regardless of their origin (42, 92). This is because CSF is in more direct contact with brain lesions, so shedding may be more evident in this fluid by a contiguity mechanism (93). For example, Pan et al. analyzed cfDNA samples from plasma and CSF of 7 patients with brain tumors, including metastatic lesions, using a combined digital PCR (polymerase chain reaction) and sequencing strategy and found a higher concentration of somatic mutations characteristic of primary tumors such as *EGFR* or *PI3KCA* in the CSF (94). Of note, a postmortem analysis of patients with BCBM in which ctDNA was evaluated in CSF identified truncal mutations from the tumors of origin but also variants unique to the brain lesions, highlighting the potential for diagnosing this type of lesion in breast tumors (92). Using targeted sequencing, Liang et al. analyzed the somatic variants present in CSF from 21 patients with glioma and 7 patients with cerebral disease with primaries located mainly in the lung and digestive tract (95). The results of the study showed that the genes altered in primary and metastatic brain tumors are different. Specifically, the ctDNA of metastatic brain lesions was characterized by the presence of alterations in *ALK* and *MDM2*. The authors suggest that this differential profile could be of interest for the diagnosis and therapy of BM (95).

Rubio-Pérez et al. characterized the immune composition of brain lesions and matched CSF by means of single-cell RNA sequencing and also genotyped the T cell receptors+ (96). The results obtained showed that tumor immune infiltration and specifically CD8^+^ T cell infiltration can be assessed in CSF samples (96). Consistently, the same T cell receptor clonotypes were present in brain lesions and CSF, demonstrating immune cell exchange between the tissue and the CSF (96). Overall, these data support the use of CSF to analyze the immune composition in a minimally invasive sample and represent a promising tool to anticipate the response to ICI (96).There is another in which single cell RNA sequencing (scRNA-Seq) was performed to characterize cell types in CSF from 19 patients with leptomeningeal metastases from different solid tumors, mostly breast cancer, who received treatment with ICIs, found a significant increase in CD8^+^ T lymphocyte population and higher levels of IFN-γ after treatment in the patients who obtained the greatest clinical benefit (97).

As it was previously commented, there are currently active treatments to treat metastatic brain lesions such as immunotherapy or targeted therapies, such as *ALK, MEK, BRAF* or *HER2* inhibitors. Studies have shown that clinically relevant variants in these genes can be detected by studying ctDNA in CSF from lung, melanoma, or breast tumors, among others (92, 94, 98). As with these targeted therapies, the study of ctDNA in CSF samples has shown value in determining the tumor mutational burden (TMB) which, as mentioned above, is a predictive biomarker of response to immunotherapy (89). Thus, Guao et al. described a high correlation between TMB measured in tumor DNA present in CSF and tumor tissue of patients with gliomas, and the result may be transferable to the characterization of BM (99).

Despite the lower concentration of ctDNA in the blood released by cerebral metastatic lesions, several studies have demonstrated the possibility of genotyping these lesions using this strategy. For example, in patients diagnosed with advanced lung cancer with BM, *EGFR* status has been successfully characterized in plasma ctDNA, showing good levels of concordance with the original primary tumor (100). Also, in plasma DNA from BC patients with brain lesions, Pangeni et al. have recently demonstrated the presence of differentially methylated markers (CTD-2028 M8, CCDC8, miR3193 and miR124-2) already present in primary tumors, showcasing that plasma can provide valuable information also in this patient profile (101). On the other hand, in the BREAK2 clinical trial, patients with *BRAF*-mutated metastatic melanoma were evaluated for response to oral dabrafenib. This study included a comparative analysis of *BRAF* status in cfDNA in plasma and tissue. Data obtained from plasma analysis showed good concordance with tissue and predictive value for response, including patients with brain lesions (102).

In addition to point mutations, numerous studies have demonstrated the possibility to analyze both TMB and MSI in plasma cfDNA and select immunotherapy in patients with advanced tumors regardless of metastatic location, although it is important to determine the possibility of false negatives (90, 103). ctDNA monitoring may be helpful in distinguishing progression during immunotherapy treatment, although no studies have addressed this utility particularly in patients with BM (104, 105). Exceptionally, in a study published by Lee et al. the role of ctDNA in monitoring melanoma patients with BM treated with anti-PD1 immunotherapy was examined (106). They analyzed *BRAF*, *NRAS* mutations and *c-KIT* in cfDNA in serial plasma samples during the first 12 weeks of treatment and assessed both intracranial and extracranial disease using the RECIST criteria. Of the 72 patients included, 13 had intracranial metastases exclusively and 59 had intra and extracranial metastases. Detection of ctDNA was 0% and 64%, respectively (106). Detection of ctDNA in plasma was associated with disease volume, while absence of ctDNA detection during treatment was associated with extracranial but not with intracranial response (106). The median OS in those patients with undetectable ctDNA versus detectable ctDNA at baseline was 39.2 and 10.6 months, respectively (106). These results suggest that ctDNA could be considered a strong prognostic biomarker in patients with melanoma and cerebral disease, especially in the subgroup with extracranial disease. In addition, our research group demonstrated the value of monitoring total cfDNA levels in 46 patients with non-small cell lung cancer who received anti-PD1 treatment but only 3 patients in this cohort had cerebral metastatic lesions (107).

### 5.2 Circulating tumor cells

The development of metastasis is a multistep procedure during which tumor cells must gradually detach from the primary tumoral lesion and locally occupy the stroma and surrounding tissues to reach the circulatory or lymphatic system. At this point, intravasated tumor cells or CTCs represent a valuable element in understanding the process of malignant dissemination but also as a reflection of tumor burden and the dynamic evolution of tumors (108, 109). The low proportion of CTCs in the bloodstream together with the molecular heterogeneity that characterizes these cells is the principal challenge for CTCs isolation and detection. All technologies isolate these cells focusing on differential features between CTCs and blood cells, such as protein expression, morphology, and biophysical properties. These technologies can be categorized based on the method of isolation as antigen-dependent or antigen-independent (110). The most employed strategy is usually carried out using antibodies that recognize epithelial cell adhesion molecule (EpCAM) conjugated with magnetic nanoparticles (111). Among the current EpCAM-based technologies, CellSearch^®^ system is the “gold standard” for the CTC detection methods (112). Antigen-independent methods are based on the physical properties of CTCs such as density, electric charges (DEP, dielectrophoresis), size and deformability, among others. The principal advantage in comparison with the antigen-dependent methods is the isolation of CTCs with a low epithelial phenotype. Size-based methods are the most common antigen independent strategies. They are since tumor cells are larger than blood cells and they can be isolated using filter-based strategies such as ISET assay microfluidic chips, Parsortix system and methods based on centrifugal forces (56, 113, 114).

Independently of the isolation strategy, CTCs main clinical utility has been demonstrated in patients with advanced neoplasia where its validity as a prognostic biomarker has been demonstrated in numerous studies with different solid tumors, such as breast, colon, prostate, or lung cancer (115). In addition to its value as a prognostic and monitoring biomarker, the molecular characterization of the population of CTCs present in different biofluids is a very useful tool when tumor lesions are not accessible, as in the case of BM. This molecular characterization has provided a better comprehension of the mechanisms that facilitate the appearance of BM. Thus, for example, analyzing CTCs from lung cancer patients in comparison with the original tumor and metastatic disease describes the appearance of mutations in genes involved in the response to cellular stress, such as *Keap-1*, *Nrf2* and *P300*, which are key players in the Keap1-Nrf2-ARE signaling pathway and which could provide a survival mechanism for CTCs when they are in the process of distant tumor dissemination (116).

On the other hand, Boral et al. characterized the population of CTCs associated with the presence of BM in patients with advanced breast cancer (117). This study identified the activation of an important Notch signaling pathway, which is associated with the appearance of brain lesions, as well as the identification of new inflammatory and immunomodulatory networks, whose role in immune evasion could favor the appearance of brain metastases (117). Prior to this work, the same research group associated the existence of a *HER2^+^*/*EGFR^+^*/*HPSE^+^*/*Notch1^+^* population in tumor lines generated from patient CTCs with the generation of BM (118).

Another relevant study characterizing CTCs from patients with advanced breast cancer identified cyclooxygenase *COX2* (PTGS2), the epidermal growth factor receptor (*EGFR*) ligand HBEGF and the alpha2,6-sialyltransferase ST6GALNAC5 as key players for tumor cells to cross the blood-brain barrier (119). Finally, also in breast cancer, the characterization of CTCs in the subtype named triple negative breast cancer patients, which frequently metastasize to the brain, identified HER2 positivity as a hallmark of tumor progression (120).

In addition to the study in blood, the presence of CTCs has been described in CSF in patients with brain dissemination. For example, Ruan et al. established an efficient procedure for collecting CTCs from CSF in five patients with leptomeningeal metastases of lung adenocarcinoma and characterized the CTCs at the transcriptomic level (121). These cells showed increased expression of genes regulating cell adhesion mechanisms such as MMP7, CLDN7 and ICAM1, as well as genes associated with a primarily epithelial phenotype (121). The detection of CTCs in CSF also represents a diagnostic tool of great interest to confirm the presence of leptomeningeal disease in tumors of different origin (122). Also, Darlix et al. analyzed the presence of CTCs in 40 breast cancer patients with suspected leptomeningeal metastases obtaining a good diagnostic sensitivity (100%) and specificity (78%) (123).

Despite all these studies demonstrating the value of CTCs analysis to characterize primary tumors located at central nervous system or BM, their utility to predict or monitor the activity of immunotherapy in this tumor context is still unexplored.

### 5.3 Extracellular vesicles

Extracellular vesicles (EVs) are known to play a relevant role as communicative mediators favoring the preparation of the pre-metastatic niche, including BM (124, 125). In addition to this communicative role, the analysis of Evs levels and their molecular cargo in different body fluids represents a promising strategy to obtain a liquid biopsy from the tumor (126). Besides, tumor-secreted Evs have the intrinsic ability to breach the brain barrier and therefore can participate in the brain colonization by cells originated from other tumor location (127).

Evs isolation can be addressed with different strategies based on their biochemical composition, size, and density, among other properties (128). The most employed strategies are ultracentrifugation, ultrafiltration, and immunoaffinity capture, size exclusion chromatography and density gradient separation. Ultracentrifugation employees high speed centrifugation to separate the Evs while size exclusion chromatography and density gradient isolation separate Evs from other debris. Affinity-based methods for EV isolation are known to produce highly specific isolation results. Immunoaffinity strategies are based on the use of antibody-coated beads to specifically recognize surface antigens (129, 130). The selection of the isolation method should be mainly made based on the sample requirements for downstream analyses.

Taking this into account the study of Evs in plasma or CSF from patients with BM has gained interest in the last years. Thus, a recent work by Carretero-González et al. compared the levels of plasmatic Evs in patients with different solid tumors with or without BM and described lower number of cEVs levels while higher protein concentration in patients with brain affectation (131). In particular, melanoma patients with cerebral disease have decreased *STAT3* activation and increased PD-L1 content in plasma Evs. In this sense, release of Evs PD-L1^+^ has been described as a resistance mechanism in patient treated with immune checkpoint inhibitors, thus the presence of Evs PD-L1^+^ in patients with BM may potentially have an impact on the response to immunotherapy (132).

In addition to the cEV levels or the total Evs protein content different molecules have been described in plasma cEVs as potential biomarkers or biological mediators in tumors with brain dissemination. For instance, different miRNAs contained in cEVs have shown to be specifically increased in tumors with BM. Thus, Wei and collaborators described the presence of MicroRNA-550a-3-5p in plasma Evs from lung cancer patients as an important factor promoting BM thanks to the direct regulation of *YAP1* (133). Also in lung cancer patients, the characterization of Evs associated miRNAs present in CSF samples served to identify miR-34b-3p and miR-335-5p as specially expressed in lung cancer patient with brain dissemination (134). Also, in CSF high levels of miR-200 family members were found in BC patients with brain secondary disease (135). In a recent study, Catelan et al. compared a panel of genes in serum exosomes isolated in tumor with brain dissemination and high-grade gliomas, finding a common increase of miR-21 levels in both cancer groups in comparison with healthy controls and a specific increment of miR-124-3p in the tumors with brain dissemination (136). Another non-coding RNAs such as LncRNA XR_429159.1 or LncRNA GS1-600G8.5 expressed in Evs were reported as relevant for the brain barrier disruption in the context of SCLC and BC (137, 138). Also, *XIST* LncRNA has been described associated with brain dissemination in BC patients. It was found to be downregulated in BM and this downregulation was associated with increased exosomal miR-503 secretion, which regulates microglia polarization and suppresses T cell growth (139).

All these data exemplify the interest of cEVs in the context of BM to better understand the mechanisms favoring brain colonization but also in the modulation of the response to therapy, especially in the context of immunotherapy.

## 6 Conclusions and future directions

In recent decades, solid tumors have made significant advances in the treatment of advanced disease, increasing survival and quality of life. New treatments such as immunotherapy and targeted therapy are the cause of this improvement. However, there are still scenarios, such as metastatic disease at the brain level, which remain a challenge due to high morbidity and mortality. Traditionally, antineoplastic drugs do not penetrate brain tissue because of the blood-brain barrier. In this review, we summarize results obtained in patients with brain disease recruited in the principal studies evaluating immunotherapy strategies, who represent a small cohort of patients due to the low representation of these patients in phase III clinical trials and the limited number of specific studies in this population. Derived from this factor, the present review has the limitation of the small number of patients, the heterogeneity of the population which includes limited and extensive cerebral disease and diverse types of primary tumors. In addition, we noticed that this group of patients is not always described in the articles, which makes it difficult to extrapolate to clinical practice. Besides is important to mention the fact that our work is not a systematic review, but a narrative comprehensive qualitative study. Although these limitations the review highlights the benefit of using immunotherapy to treat breast, lung, and melanoma tumor with BM, opening new therapeutic options to manage disseminated disease. However, immunotherapy has some serious toxicity that must be monitored and treated. Therefore, it is crucial to be able to accurately select patients who will benefit most of the immunotherapy at brain level and avoid endangering vital organs.

In this clinical context, the application of liquid biopsy may have special relevance, as a tool that avoids invasive procedures and provides molecular information in real time for the monitoring of the disease throughout its evolution. As it was detailed in this review, there is evidence supporting the utility of liquid biopsy analyses for immunotherapy selection and monitoring in lung, breast, and melanoma cancers. Combined use of immunotherapy and liquid biopsy have a proven benefit, making them important achievements in the therapeutic and diagnosis arsenal of the mentioned tumors. Several works have demonstrated that liquid biopsy analysis (mainly CSF and blood markers) in the context of BM from breast, lung, and melanoma tumors, allow to molecularly characterize the disease taking into account the tumor evolution and heterogeneity and determine the status of well stablished immune biomarkers such as PD-L1, TMB or MSI condition. In this sense, cfDNA analyses in CSF have demonstrated special value to interrogate the tumors located in CNS, including BM. However, our review showed the general lack of information about the specific utility of the cfDNA assessment and other markers such as CTCs and EVs in patients with BM receiving immunotherapy. In this regard, it is important to mention that all the phase III trials described in advanced tumors (NSCLC, SCLC, breast, melanoma) were not associated with translational studies, so there is no analysis of liquid biopsy that could serve as secondary endpoints. This makes it difficult to draw conclusions about the behavior of circulating biomarkers during immunotherapy treatment, but at the same time it creates the need and challenge to improve the design in future clinical trials focused on a subgroup of patients of particular concern due to their poor prognosis. Of note, the present review englobes for the first time the results regarding the combined value of immunotherapy and liquid biopsy in the context of BM and provides comprehensive knowledge that might open the door to the creation of specific clinical trials to interrogate value of circulating biomarkers for the diagnosis, prognosis, and monitoring of the response to immunotherapy in patients with brain lesions. Fortunately, there are ongoing studies and preliminary results in lung, breast, and melanoma cancer, which aim to provide more molecular information in this setting to better meet the needs of this subgroup of patients. Further research is warranted to make progress in the application of immunotherapy to treat solid tumors with BM and liquid biopsy to guide the treatment in a cohort of patients with poor therapeutic alternatives.

## Author contributions

All authors performed the literature review, collected all data, and wrote the manuscript. All have read and approved the final manuscript.
